# EV71 virus-like particles produced by co-expression of capsid proteins in yeast cells elicit humoral protective response against EV71 lethal challenge

**DOI:** 10.1186/s13104-015-1780-x

**Published:** 2016-01-25

**Authors:** Xiaowen Wang, Xiangqian Xiao, Miao Zhao, Wei Liu, Lin Pang, Xin Sun, Shan Cen, Burton B. Yang, Yuming Huang, Wang Sheng, Yi Zeng

**Affiliations:** College of Life Science and Bioengineering, Beijing University of Technology, 100 Pingleyuan, Chaoyang District, Beijing, People’s Republic of China; Department of Neurology, Beijing Ditan Hospital, Capital Medical University, Beijing, People’s Republic of China; Research Center for Life Science, Beihua University, Jilin, People’s Republic of China; Department of Virology, Institute of Medicinal Biotechnology, Chinese Academy of Medical Science, Beijing, People’s Republic of China; Department of Laboratory Medicine and Pathobiology, University of Toronto, 2075 Bayview Avenue, Toronto, M4N 3M5 Canada

**Keywords:** Enterovirus 71, Vaccine, Yeast, Virus-like particle, Hand foot and mouth disease

## Abstract

**Background:**

Enterovirus 71 (EV71) is the most common causative pathogens of hand, foot and mouth disease (HFMD) associated with severe neurological complications. There is a great need to develop prophylactic vaccine against EV71 infection.

**Results:**

EV71 virus-like particle (VLP) was produced in yeast expression system by the co-expression of four EV71 structural proteins VP1–VP4. Immunization with the recombinant VLPs elicited potent anti-EV71 antibody responses in adult mice and anti-VLP sera were able to neutralize EV71 virus in vitro. Neonatal mice model demonstrated VLP immunization conferred protection to suckling mice against the lethal viral challenge.

**Conclusions:**

Co-expression of four EV71 structural proteins VP1–VP4 in yeast expression systems is an effective method to produce EV71 VLPs. VLP-based vaccine shows great potential to prevent EV71 infection.

## Background

Human enterovirus 71 is a non-enveloped RNA virus of the Picornaviridae family that was first reported in 1969. The virion is around 25–30 nm in diameter containing a single-stranded RNA viral genome of approximately 7500 nucleotides [1–3]. EV71 encoded a single large polyprotein that is initially cleaved into P1, P2, and P3 regions. The P1 region is subsequently processed by protease 3CD to generate four capsid subunit proteins, VP1 to VP4. The viral genome is packaged in an icosahedral capsid which is composed of 60 copies of structural proteins. High-resolution structural analysis showed that VP1-3 form a pseudo T = 3 icosahedral capsid that are located on the surface of viral capsid [4]. VP4 is myristoylated at the N terminus and located inside virion [5, 6]. However, crystallographic analysis demonstrated that the structure of mature EV71 virion is similar to other enteroviruses [7].

EV71 has been identified as one of the major etiological agents of hand, foot and mouth disease (HFMD) [8, 9]. A number of HFMD epidemics caused by EV71 infection occurred in the Asia–Pacific region and are associated with severe neurological complications such as aseptic meningitis, poliomyelitis-like paralysis and brainstem encephalitis. The surveillance data from National Center for Disease Control and Prevention showed that more than 3 million HFMD cases and 1384 deaths were reported by the end of 2010 in mainland China [10–14]. HFMD is becoming the most common viral disease in these areas that seriously threat children health. However, no appropriate vaccine is yet available to prevent EV71 infection.

Vaccination is the most optimal way to prevent and reduce prevalence of viral infectious diseases. Neutralizing antibody plays an essential role in the protection of suckling mice against EV71 infection, because immunization of maternal mice with inactivated EV71 can confer protection to neonatal mice against EV71 lethal challenge [15]. Several linear immunodominant epitopes were screened and successfully identified in EV71 structural proteins [16–20]. In recent years, recombinant virus-like particle (VLP)-based vaccine strategies have been frequently used for novel vaccine design. Vaccines based on viral VLPs have been successfully used in prevention against hepatitis B virus and human papillomavirus [21–23]. VLPs are empty non-infectious viral capsids that structurally mimic the conformation of native virion. High density of viral linear and conformational epitopes on the VLP surface may elicit strong immune responses [23]. In the present study, EV71 VLPs were successfully produced by co-expression of four structural viral proteins in yeast, which is safe, reliable and cost-effective platform for recombinant protein production.

## Results

### Generation and characterization of EV71 VLPs

**Fig. 1:**
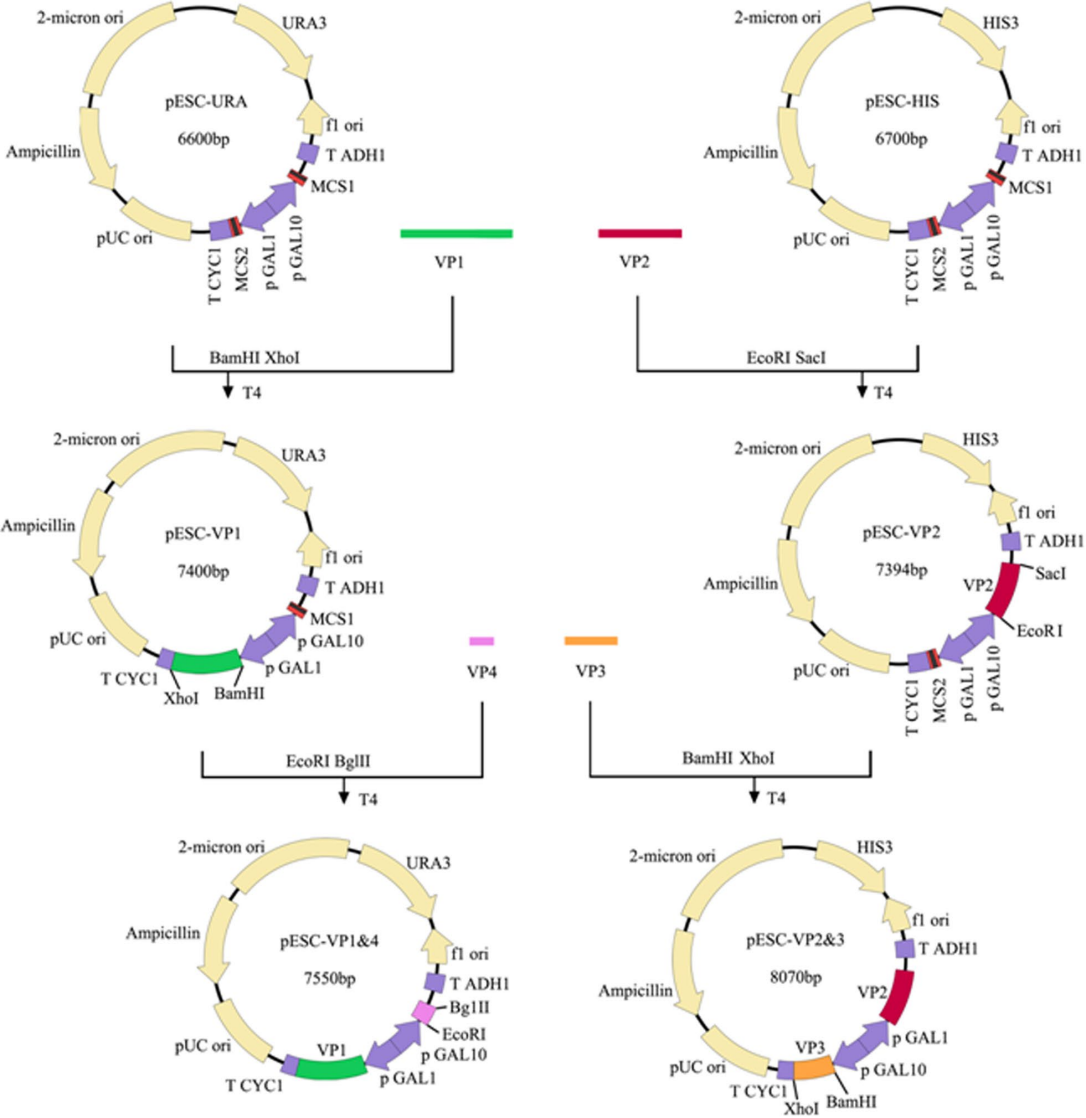
Schematic illustration of the construction of recombinant plasmids

**Fig. 2:**
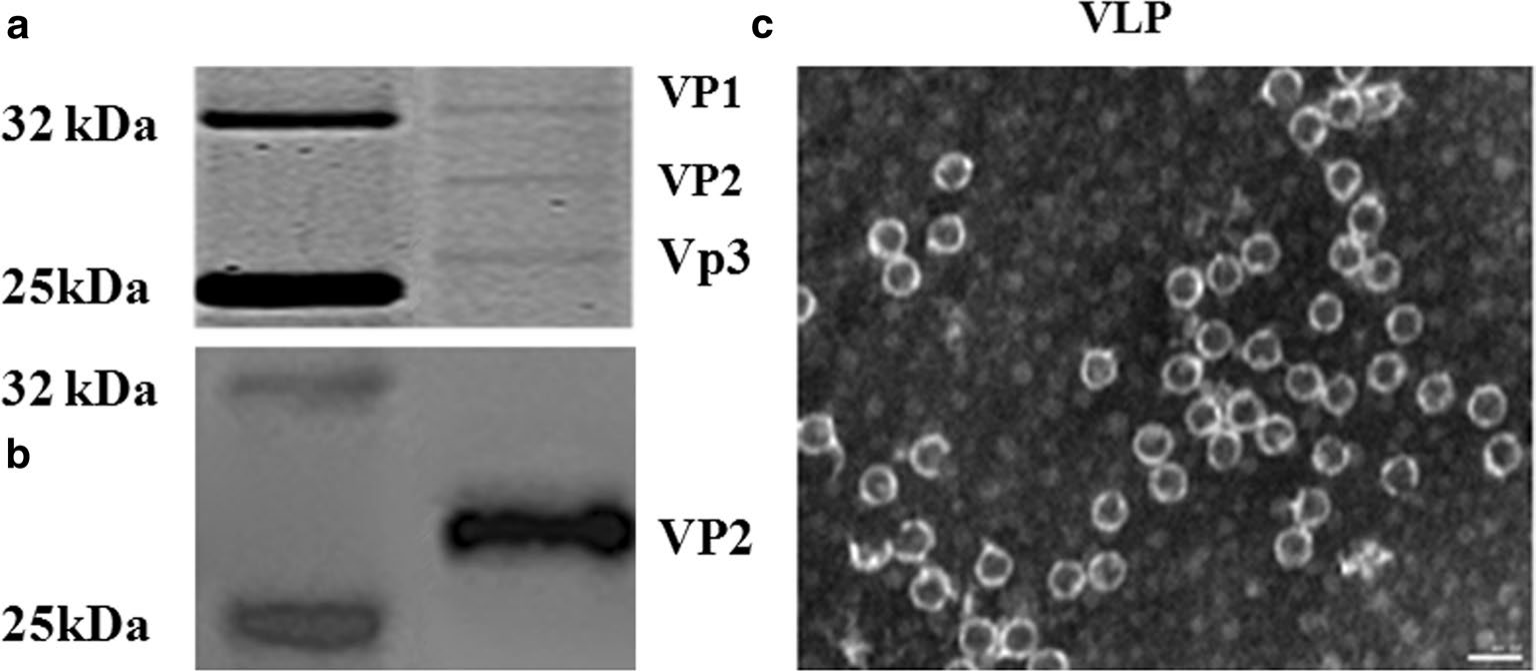
Viral protein expression and electron microphotographs. **a** The expressions of EV71 structure proteins were visualized by SDS-PAGE. **b** The expression of EV71 VP2 was monitored using VP2-specific antibody MAB979 by Western blot. EV71 VLPs were isolated using sucrose and discontinuous cesium chloride (CsCl) gradient (1.4, 1.33, 1.29 and 1.25 g/ml) by ultracentrifugation. The formation of EV71 VLPs in yeast was monitored by electronic microscope. *Size bar* 50 nm

EV71 capsid is composed of 60 copies of each of the four viral structural proteins VP1, VP2, VP3 and VP4. In the present study, genes encoding VP1, VP2, VP3 and VP4 proteins of EV71 were inserted into vectors for co-expression of four viral proteins in yeast (Fig. 1). The expressions of viral proteins were investigated by SDS-PAGE and Western-blot. Viral proteins were purified using the method described previously with modification [3] and visualized using SDS-PAGE analysis. Three bands were observed which corresponded to VP1, VP2 and VP3 of EV71 virus, respectively, according to the molecular weight of each band (Fig. 2a). However, the band corresponding to EV71 VP2 protein was further proofed by using VP2-specific antibody MAB979 by Western-blot (Fig. 2b). To determine whether the co-expression of four viral structural proteins can generate EV71 VLPs, total proteins of yeast cells were extracted and loaded onto the sucrose and cesium chloride gradient to isolate EV71 VLPs by ultracentrifugation. As shown in Fig. 2c, the formation of viral VLPs were observed in purified yeast lysates by transmission electron microscope and the diameters of particles were about 25–27 nm which appeared icosahedral. Our data indicate that co-expression of four viral structural proteins VP1, VP2, VP3 and VP4 in yeast cells can definitely lead to the formation of EV71 VLPs.

### VLP immunization elicits neutralizing antibody responses against EV71

**Fig. 3:**
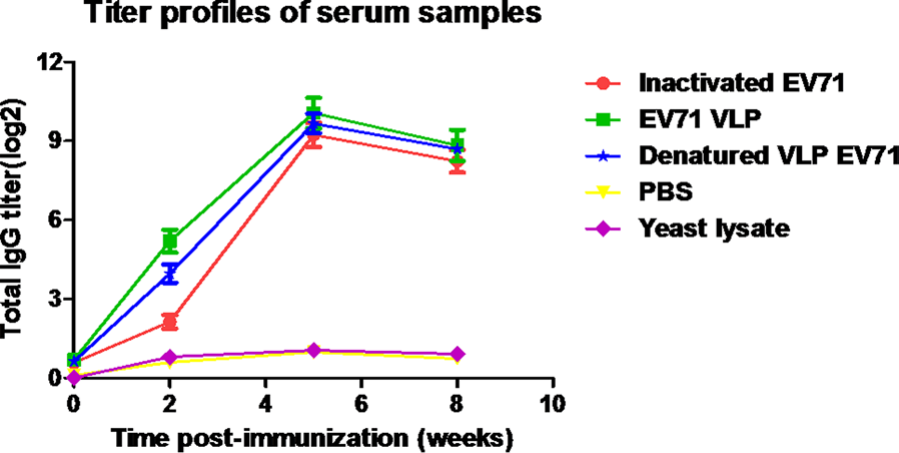
Kinetics of antibody titer development in mice following immunization. The inactivated EV71 virus was coated on the plate surface to capture anti-EV71 antibody. The data were represented by the mean of reciprocal log^2^ endpoint titers ± standard deviation (SD)

To investigate whether the EV71 VLPs were capable of inducing anti-EV71 antibody responses, female BALB/c mice were immunized i.m. with either purified VLPs, denatured VLPs or inactivated EV71 virus at the dose of 20 μg/mouse and received booster injections 3 weeks later. Mice immunized with yeast cell lysates and PBS were used as negative controls. The immunized animals were bled at week 0, 2, 5, 8 for serological analysis. The results showed that anti-EV71 IgG antibodies became detectable in mice immunized by EV71 VLPs, denatured VLPs and inactivated-EV71 virus at 2 weeks after inoculation. The titers were enhanced by booster injection and reached a maximum titration at week 5. No anti-EV71 antibody response was detected in the yeast cell lysates-immunized group and the PBS group (Fig. 3). Our results demonstrated that EV71 VLPs, denatured VLPs and inactivated-EV71 virus induced similar levels of anti-EV71 antibody.

**Fig. 4:**
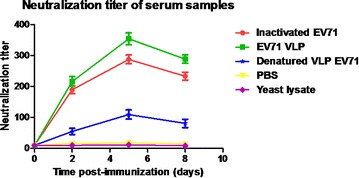
Kinetics of neutralizing antibodies to EV71 following immunization. In vitro microneutralization assay was used to detect neutralizing antibodies in the sera from immunized mice. The neutralizing antibody titer was defined as the highest serum dilution that prevented the occurrence of cytopathic effects. The data was represented by the mean of endpoint titers ± SD

**Fig. 5:**
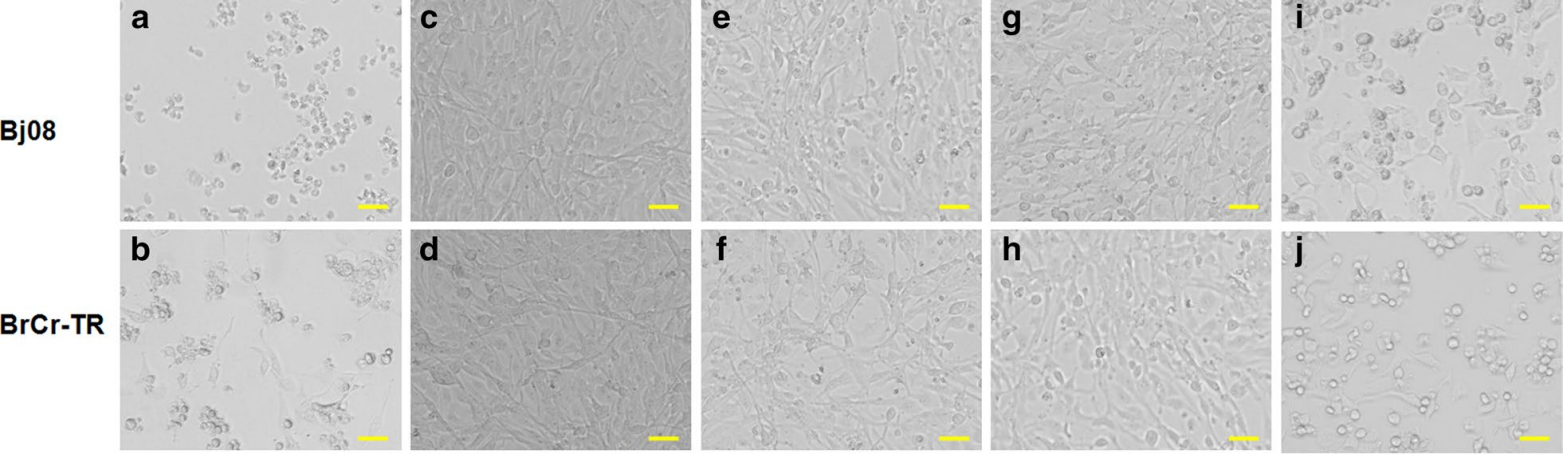
In vitro neutralization assay. **a** Cells infected by EV71 Bj08 strain. **b** Cells infected by EV71 BrCr-TR strain. **c**, **d** Uninfected cells. **e** Cells infected by Bj08 strain reacted with anti-VLP serum. **f** Cells infected by BrCr-TR strain reacted with anti-VLP serum. **g** Cells infected by Bj08 reacted with anti-inactivated EV71 serum. **h** Cells infected by BrCr-TR strain reacted with anti-inactivated EV71 serum. **i** Cells infected by Bj08 reacted with anti-denatured VLP serum. **j** Cells infected by BrCr-TR strain reacted with anti-denatured VLP serum. *Size bar* 50 μm

To evaluate whether the antibodies were able to neutralize EV71 virus, mouse serum samples were twofold serially diluted and mixed with infectious EV71 (100 TCID_50_) to test their ability to neutralize the live EV71 virus in vitro. EV71 Bj08 (genotype C4) and a variant of the prototype strain of EV71, BrCr-TR (genotype A) were used for in vitro neutralization assay. As shown in Fig. 4, remarkable increase in anti-EV71 neutralizing antibody titers was observed after the booster injection at 3 weeks post-injection. The EV71 VLPs elicited slightly higher neutralization titers against EV71 Bj08 compared to the heat-inactivated EV71. The denatured VLPs elicited lower neutralization titers than the purified VLPs, although they induced similar levels of IgG antibodies. These data are consistent with the previous report [3]. In vitro inhibition of EV71-mediated cytopathic effect (CPE) by anti-sera was illustrated by microscopic photographs. Serum samples were diluted 256-fold for neutralization assay. Virus-mediated CPE was remarkably found in cells infected by either EV71 Bj08 or BrCr-TR (Fig. 5a, b) after 6 days of infection. In contrast, no obvious CPE was observed in the cells without viral infection (Fig. 5c, d). Virus-induced CPE could be significantly suppressed by the addition of anti-VLP sera (Fig. 5e, f) and anti-inactivated EV71 sera (Fig. 5g, h). Lower suppression of CPE was observed by using anti-denatured VLP sera (Fig. 5i, j), suggesting that antibody recognizing conformational viral epitopes are essential for VLP-induced immune protection against EV71.

### In vivo protection against lethal viral challenge in neonatal mouse model

**Fig. 6:**
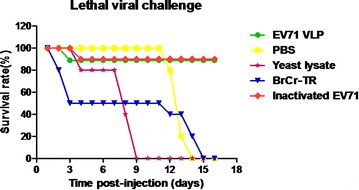
Suckling mouse was used as a model to assess neutralizing effects of anti-sera. Groups of 1-day-old BALB/c suckling mice were inoculated intraperitoneally (i.p.) with the mixture of virus and anti-sera. Survival rates were recorded daily after infection for 16 days. 10 mice were used for each group

The in vivo protective ability of anti-sera against lethal EV71 challenge was evaluated in a neonatal mouse model and EV71 BrCr-TR was used as a representative high-virulence strain. 10 LD_50_ of BrCr-TR virus was mixed with sera from mice immunized with EV71 VLPs, inactivated-EV71 virus, yeast cell lysates and PBS. The mixture of EV71 virus and anti-sera was incubated overnight at 37 °C and used to inoculate intraperitoneally (i.p.) 1-day-old BALB/c suckling mice (n = 10 per group). Infection by BrCr-TR viral strain was used as positive control. The data showed that 90 % of mice treated with EV71 virus mixed with the EV71 VLPs- and inactivated-EV71 virus-immune sera remained healthy and survived until the end of the experiments. In contrast, mice inoculated with virus mixed with the yeast cell lysates and PBS-immune sera died at 16 days post-inoculation, indicating that EV71 VLPs and inactivated EV71 virus conferred immune protection against EV71 lethal challenge in vivo (Fig. 6).

## Discussion

Vaccination is a commonly used and cost-effective method for infectious disease control. Various types of vaccine candidates against EV71 have been evaluated in animal model, including recombinant subunits [15, 24], synthetic peptide vaccines [18–20], live attenuated vaccines [25, 26], VLP vaccines [3, 27, 28], DNA vaccines [29] and formalin-inactivated virion vaccines [30–33]. Recombinant subunit vaccines can be produced from different expression systems. It has been found that EV71 capsid proteins VP1, VP2 and VP3 are immunogenic and capable of eliciting antibodies that recognize their corresponding viral proteins. Unlike VP2 and VP3, recombinant VP1 was capable of eliciting effective neutralizing antibody responses against EV71 [34]. Synthetic peptides containing neutralization epitopes are considered as good vaccine candidates providing well-defined immunogens and safety advantages. Two linear neutralizing epitopes SP70 and SP55 found in EV71 VP1, were capable of inducing neutralizing antibody responses in mice against EV71 virus by inhibiting viral infection at pre- or post-attachment steps [35]. Our previous study showed that immunization of N terminus of EV71 VP4 elicits cross-protective antibody responses [36]. Recently, a cross-neutralizing epitope was identified in EV71 VP2 [37]. However, neutralizing antibody responses induced by peptides are usually weak and require strong adjuvants. Live attenuated vaccines are widely used for viral disease prevention, however, the emergence of pathogenic viral revertants has raised vaccine safety concerns. Inactivated vaccine and VLP-based vaccine retain both linear and conformational neutralizing epitopes responsible for eliciting potent neutralizing antibody responses against viral infection [38] and are thus widely accepted as effective vaccine candidates. VLP-based vaccine has a safety advantage over inactivated vaccine due to the lack of the viral genome.

Recombinant VLPs that mimic the conformation of authentic native viruses can be produced from variable expression systems such as insect cells, yeast and *E. coli*. EV71 VLPs have been successfully produced in variable expression systems via co-expression of P1 and 3CD viral proteins [28, 39]. 3CD is an EV71-encoded protease responsible for the digestion of P1 polyprotein into structural subunit proteins, which form EV71 capsids via self-assembly.

Two types of EV71 viral particles (E- and F-particles) have previously been produced from Vero cells cultured in a serum-free condition. SDS-PAGE and Western blot analysis illustrated that the E-particle was an immature particle containing incompletely cleaved VP0 protein, while in F-particle, VP0 protein was fully cleavaged into VP2 and VP4 by autocatalytic processing. In addition, neutralization assay showed that the inactivated F-particle induced a more potent neutralizing antibody response against EV71 in the mouse model than the E-particle [40]. Thus, F-particle of EV71 is an ideal vaccine candidate to prevent EV71 infection. In the present study, we report a technique for production of EV71 F-particles in yeast cells. TEM analysis showed that EV71 VLPs were successfully generated in yeast cells through the co-expression of four EV71 capside proteins (VP1 to VP4) without using 3CD protease. It has been reported that host cell apoptosis can be triggered by the coxsackievirus B3-encoded protease and HIV-encoded proteases induced cell death [41, 42]. Co-expression of P1 protein and 3CD can be used to produce EV71 VLP in different host cells. However, the effects of EV71-encoded protease on host cell apoptosis or death still remains to be elucidated.

EV71 is phylogenetically classified into three distinct genotypes: A, B, and C based on the genetic variation of VP1 sequences. Genotypes B and C can be further divided into B1–B5 and C1–C5 subgenotypes, respectively [43, 44]. Recently, 3 new genotypes were reported, D, E and F subgenotypes [45, 46]. An ideal anti-EV71 vaccine should be able to provide broad protective activity against a wide spectrum of EV71 strains. One single serotype was reported in EV71 when measured by hyperimmune animal sera. However, antigenic variations have been identified recently in post-infection human sera when measured cross-neutralizing antibody titers against different EV71 genotype strains. Antigenic variation among different genogroups was reported [47]. Immunization of live attenuated EV71 vaccine (genotype A) induced the strongest neutralizing antibody responses against the homotype (genotype A) and weaker neutralizing antibody responses against other EV71 genotype strains such as B1, B4, C2 and C4, suggesting antigenic heterogeneity among EV71 isolates [26]. Five EV71 vaccine candidates are currently being evaluated in clinical trial. Genotype C4 and B4 viruses were selected for anti-EV71 vaccine development in mainland China and Taiwan, respectively. The inactivated EV71 C4 genotype vaccine could provide homogeneous and broad cross-protection against different subgenotypes of EV71 such as B4, B5, C2, C4 and C5 strains in health children and infants [48]. An potent cross-neutralizing antibody protection against EV71 subgenotypes B1, B4, B5 and C4A were also observed in B4-based vaccine treatment [49]. Based on the antigenic heterogeneity among EV71 isolates and the efficacy of multivalent VLP vaccine demonstrated by the successful application of bivalent and tetravalent HPV vaccine, multivalent VLP-based vaccine has a great potential in the development of a safe and cost-effective anti-EV71 vaccine with broad cross-protection.

## Conclusions

The co-expression of four EV71 capsid proteins in yeast resulted in effective production of EV71 VLPs, which elicited potent anti-EV71 antibody responses and protected neonatal mice against lethal viral challenges. Multivalent EV71 VLP-based vaccine has a great potential to be a safe and cost-effective vaccine candidate with broad cross-neutralizing activities against EV71 infection.

## Methods

### Construction of plasmids

The coding sequence of EV71 P1 protein of C4 subgenotype was optimized according to the codon usage for *S. cerevisiae*. DNA fragment encoding P1 protein was then synthesized. VP1 gene was amplified using primers VP1-BamH1 (5′-AAGGATCCATGGGTGACAGAGTCGCCGAT-3′) and VP1-*Xho*I (5′-CCGCTCGAGTCATAAAGTAGTGATGGCT-3′. The PCR products were double-digested by *Xho*I and *Bam*H1 and subsequently inserted into the vector pESC-URA (Invitrogen) to construct pESC-VP1. VP4 gene was amplified using primers VP4-*Eco*R1 (5′-CCGGAATTCATGGGATCACAAGTTTCA-3′) and VP4-*Bgl*II (5′-GAAGATCTCTTTAATGGGGCAGCCAT-3′). The PCR fragments were further inserted into the vector plasmid pESC-VP1 at *Eco*R1 and *Bgl*II sites to yield pESC-VP1&4. VP2 gene was amplified using primers VP2-*Eco*RI (5′-CGGAATTCATGTCTCCATCTGCCGAAGCA-3′) and VP2-*Sac*I (5′-AACGAGCTCCTGAGTAACGGCTTGCCT-3′). The amplified PCR products were then inserted into the vector pESC-HIS (Invitrogen) to construct pESC-VP2 after double-digestion by *Eco*R1 and *Sac*I. VP3 gene was amplified using primers VP3-*Bam*HI (5′-CGGGATCCATGGGTTTTCCTACAGAATTG-3′) and VP3-*Xho*I (5′-CCGCTCGAGTTGTATTATTCCAGTCTG-3′). The amplified DNA fragments were then inserted into the plasmid pESC-VP2 at *Bam*H1 and *Xho*I cutting sites to yield pESC-VP2&3.

### Expression of recombinant protein in yeast

The recombinant plasmids pESC-VP1&4 and pESC-VP2&3 were further co-transformed into yeast of INVSc1 strain (Invitrogen, USA) by using Sc EasyComp transformation kit (Invitrogen, USA) according to the instructions of the manufacturer. The yeast transformants were further selected onto a synthetic complete plates without uracil and histidine. Clonal isolates were then grown at 30 °C for 78 h in YPD containing 2 % galactose to an optical density. After centrifugation, the harvesting cell pellets were broken with glass beads and cell lysates were analyzed for the expression of EV71 VP1.

### Purification of VLPs

The yeast pellets were resuspended in sodium phosphate buffer (pH 7.2) supplemented with 1× rotease inhibitor cocktail. Yeast cells were broken by using high-pressure homogenizer (APV-2000, Denmark) with a pressure of 1500 Bar. 25 U/ml of benzonase (Merck, USA) was further added into the cell lysates and incubated for 1 h at room temperature. Cellular debris was then removed by centrifugation at 12,000*g* for 20 min at 4 °C. NaCl was added into the supernatant at a final concentration of 500 mM and polyethylene glycol 5000 (Sigma-Aldrich, USA) was added at a final concentration of 10 % (w/v). After an overnight mixture at 4 °C, the supernatant was centrifuged at 12,000*g* for 20 min at 4 °C and the pellets were resuspended into sodium phosphate buffer (pH 7.2). Insoluble debris was removed by centrifugation at 10,000*g* for 10 min at 4 °C. The clarified supernatant was loaded onto the discontinuous sucrose gradient consisting of 15, 35 and 65 % sucrose that was dissolved in PBS buffer. After ultracentrifugation at 100,000*g* using SW32Ti rotor (Beckman, USA) for 6 h, the white band between the interfaces of 15–35 % sucrose was collected and loaded onto discontinuous cesium chloride (CsCl) gradient (1.4, 1.33, 1.29 and 1.25 g/ml) and spun using SW32Ti rotor (Beckman, USA) for 21 h at 100,000*g*. The VLP-containing fractions were harvested, diluted with sodium phosphate buffer, and ultracentrifuged at 34,000*g* for 6 h at 4 °C after dialyzed against low salt PBS buffer (pH 7.4). The pelleted VLPs were resuspended in PBS and concentration was measured using Bradford protein assay kit (Bio-Rad, USA).

### SDS-PAGE and western blotting

The purified VLP samples were denatured by boiling for 10 min and loaded onto SDS-PAGE (12 %) gel for electrophoresis. The recombinant proteins were detected by Western blotting using a monoclonal antibody against VP2 (MAB979, Millipore, USA). Briefly, the proteins were transferred onto PVDF membrane, which were blocked with 2 % (w/v) BSA in TBS solution for 1 h at room temperature, and further washed three times with TBS containing 0.05 % (v/v) Tween 20. The membrane was then incubated with primary anti-VP1 and anti-VP2 antibodies, respectively, for 1 h at 37 °C, and washed three times with TBS buffer. After incubation with the secondary goat anti-rabbit and goat anti-mouse antibodies conjugated with fluorescent dyes: IRDye 800 CW (KPL, USA) for 45 min, blotting images were acquired using the Odyssey infrared imaging system (Li-COR Biosciences, USA) and analyzed by the software provided by the manufacturer.

### Electron microscopy

The formation of EV71VLPs was analyzed by electron microscopy as described previously [3]. Briefly, samples were adsorbed to carbon-coated copper grids and incubated for 1 min. The grids were then negatively stained for 45 s with 2 % phosphotungstic acid after washing twice with PBS and visualized using an electron microscope (H-7650, HITACHI, Japan).

### Immunization of animals

Pathogen-free female BALB/c mice were purchased from Beijing HFK Bioscience Co. (Beijing, China). All animals were housed at pathogen-free conditions. Animal experiments were performed according to the current experimental protocols involving animal study approved by the Institutional Animal Care and Use Committee of Peking University. For mice experiments, five female BALB/c mice (6–8 weeks) per group were immunized with 20 μg/mouse of one of the following samples: purified VLPs, denatured VLPs, beta-propiolactone-inactivated EV71 virus (Bj08 strain), yeast cell lysate or PBS. The immunization was boosted 3 weeks later with the same dosages. QuickAntibody™ from KBQ Biotechnology Co. (Beijing, China) was used as an adjuvant. Control group was immunized with PBS plus adjuvant. The blood samples were collected at week 0, 2, 5, 8 and the sera were inactivated at 56 °C for 30 min and stored at −80 °C.

### ELISA analysis

Inactivated EV71 virus were used as the coating antigen to titrate anti-EV71 IgG levels in the serum samples by sandwich enzyme-linked immunosorbent assay (ELISA) as described previously [3]. Briefly, 96-well plates were coated with polyclonal anti-EV71 antibody overnight at 4 °C and blocked with the buffer containing 2 % (w/v) bovine serum albumin for 2 h at 37 °C. Inactivated EV71 virus was added to the well and incubated for 2 h after washing thrice with the buffer (0.05 % (v/v) Tween 20 in PBS). The sera were analyzed at twofold serial dilutions by starting at 1:100. The plates were incubated at 37 °C for 1 h and washed thrice with buffer. HRP conjugated goat anti-mouse IgG (CWBIO, China) was then added into each well in a 1:2000 dilution, and incubated at 37 °C for 1 h. The plates developed with TMB solution (Tiangen Biotech, China) in a dark room for 15 min after washing three times with buffer, and the reaction was stopped by adding 2 M H_2_SO_4_. The absorbance at 450 nm was evaluated using a microplatereader (Bio-Rad, USA).

### In vitro neutralization assay

Neutralization assay was carried out as described previously [36]. Briefly, EV71 BJ08 (genotype C4) and BrCr-TR (genotype A), were propagated in RD cells. The virus titers were determined in RD cells and expressed by the 50 % tissue culture infective dose (TCID_50_) according to the Reed-Muench method. To measure the neutralization titers, RD cells were cultured in the 96-well plates overnight until 60 % confluence. Serum samples were twofold serially diluted using Minimum Essential Medium (MEM, Gibco^®^) containing 2 % FBS and mixed with equal volume of EV71 (100 TCID_50_). After incubation overnight at 37 °C, the mixture was used to infect RD cells. The highest serum dilution, which could fully protect infected cells from developing cytopathic effects, was considered as neutralization titer.

### Mouse protection assay

The protective efficacy of the immunized sera was evaluated by in vivo infection experiments. Briefly, 50 μl of sera from mice immunized with recombinant VLPs, inactivated EV71, yeast cell lysate or PBS were incubated with 10 LD50 of EV71 BrCr-TR at 37 °C for 2 h. Groups of 1-day-old BALB/c suckling mice (n = 10 per group) were inoculated intraperitoneally (i.p.) with the mixture of virus and sera. All mice were monitored daily for the appearance of death for up to 16 days after inoculation.
